# Lessons Learned from Discontinued Clinical Developments in Duchenne Muscular Dystrophy

**DOI:** 10.3389/fphar.2021.735912

**Published:** 2021-11-01

**Authors:** Theodora Markati, Liesbeth De Waele, Urlike Schara-Schmidt, Laurent Servais

**Affiliations:** ^1^ MDUK Oxford Neuromuscular Center, Department of Paediatrics, University of Oxford, Oxford, United Kingdom; ^2^ KU Leuven Department of Development and Regeneration, Leuven, Belgium; ^3^ Department of Paediatric Neurology, University Hospitals Leuven, Leuven, Belgium; ^4^ Department of Pediatric Neurology, Center for Neuromuscular Diseases, Center for Translational Neuro- and Behavioral Sciences, University Duisburg-Essen, Essen, Germany; ^5^ Division of Child Neurology, Reference Center for Neuromuscular Disease, Centre Hospitalier Régional de Références des Maladies Neuromusculaires, Department of Pediatrics, University Hospital Liège, Liège, Belgium

**Keywords:** antisense oligonucleotides, clinical trials, duchenne muscular dystrophy, dystrophin, exon skipping, randomized controlled clinical trials, myostatin inhibition, utrophin upregulation

## Abstract

Duchenne muscular dystrophy (DMD) is an X-linked condition caused by a deficiency of functional dystrophin protein. Patients experience progressive muscle weakness, cardiomyopathy and have a decreased life expectancy. Standards of care, including treatment with steroids, and multidisciplinary approaches have extended the life expectancy and improved the quality of life of patients. In the last 30 years, several compounds have been assessed in preclinical and clinical studies for their ability to restore functional dystrophin levels or to modify pathways involved in DMD pathophysiology. However, there is still an unmet need with regards to a disease-modifying treatment for DMD and the attrition rate between early-phase and late-phase clinical development remains high. Currently, there are 40 compounds in clinical development for DMD, including gene therapy and antisense oligonucleotides for exon skipping. Only five of them have received conditional approval in one jurisdiction subject to further proof of efficacy. In this review, we present data of another 16 compounds that failed to complete clinical development, despite positive results in early phases of development in some cases. We examine the reasons for the high attrition rate and we suggest solutions to avoid similar mistakes in the future.

## Introduction

Duchenne muscular dystrophy (DMD) is an X-linked condition caused by deficiency of functional dystrophin protein. Dystrophin deficiency causes increased susceptibility of the sarcolemma to mechanical stress and results in high intracellular calcium levels, mitochondrial dysfunction, and increased production of reactive oxygen species that lead to a cycle of loss of muscle fibres, chronic inflammation, and decreased muscle regenerative capacity. Ultimately, muscle fibres are replaced by connective and adipose tissue resulting in muscle dysfunction. Patients experience progressive weakness in skeletal muscles and cardiomyopathy. Several isoforms of dystrophins are expressed in the central nervous system, and a large proportion of patients have cognitive involvement ranging from mild attention deficit to severe autism (Doorenweerd, 2020). DMD patients lose ambulation and have a shortened life expectancy. For a detailed review of DMD pathophysiology, we refer readers to the following reviews (Duan et al., 2021; Starosta and Konieczny, 2021). Standards of care includes steroids, multidisciplinary approaches and depending on the country, ataluren for nonsense mutation or exon skipping therapy for patients with eligible mutations. Standards of care and steroids extend life expectancy and the quality of life for patients with DMD (Birnkrant et al., 2018a; Birnkrant et al., 2018b; Birnkrant et al., 2018c); however, there is an unmet need with regards to treatment.

In the past decades, several compounds have been tested in preclinical and clinical studies for their abilities to restore functional dystrophin levels in the muscle cells or to alter the fate of downstream molecular pathways involved in DMD pathophysiology. Breakthroughs in biomedical research have led to the development of ASOs and gene therapy treatments that increase the levels of dystrophin. Except for the US Food and Drug Administration (FDA)-approved steroid for use in DMD (deflazacort), four other antisense oligonucleotides (ASOs) for exon skipping (eteplirsen, golodirsen, casimersen, viltolarsen) have received approval by the FDA through the accelerated approval pathway; viltolarsen has additionally received approval in Japan [^1^ (Aartsma-Rus and Krieg, 2017; Aartsma-Rus and Corey, 2020; Dhillon, 2020; Shirley, 2021)]. One more compound, the small molecule for stop codon readthrough namely ataluren, has received conditional approval by the European Medicines Association (EMA), but not the FDA (Ryan, 2014). However, the clinical development of several compounds with promising early-phase results has been halted due to disappointing late-phase results, tempering expectations in the DMD community.

The difficulty in translating results from animal models to humans, the heterogeneity of the DMD genotypes and symptoms such as cognitive involvement, the rarity of the disease, the lack of efficacious outcome measures or fully validated biomarkers are some of the most important reasons for the high attrition rate between early- and late-phase clinical development. In this article, we review data on the compounds that have failed in clinical development with published results within the last 5 years. Additionally, we present our perspective on the reasons for the high attrition rate in DMD clinical development and propose solutions.

## Methods

To identify compounds for which clinical development in the DMD population has been terminated, we performed a comprehensive review of four databases (Ovid MEDLINE, Embase, Scopus, and Cochrane) in English and French, restricted for the last 5 years (2016–2021). For a full list of the keywords used for the different databases, please refer to Supplementary Material. We selected full-text peer-reviewed publications of clinical studies of DMD (+/− Becker muscular dystrophy) patients of pharmacological interventions only. We made a list of the different compounds identified in the selected papers of our research strategy and we checked the status of their development online and on ClinicalTrials.gov.

Additionally, we searched ClinicalTrials.gov using the term “Duchenne muscular dystrophy” and filters “terminated” OR “suspended” OR “withdrawn” with no time limitation. Studies with unknown status were not included and we selected only studies of a pharmacological intervention intended to treat DMD (+/− Becker muscular dystrophy). In total, we identified 10 compounds from the literature review and another six from the ClinicalTrials.gov search whose development has been discontinued.

## Results

### Identification of Compounds with Terminated Clinical Development

**FIGURE 1:**
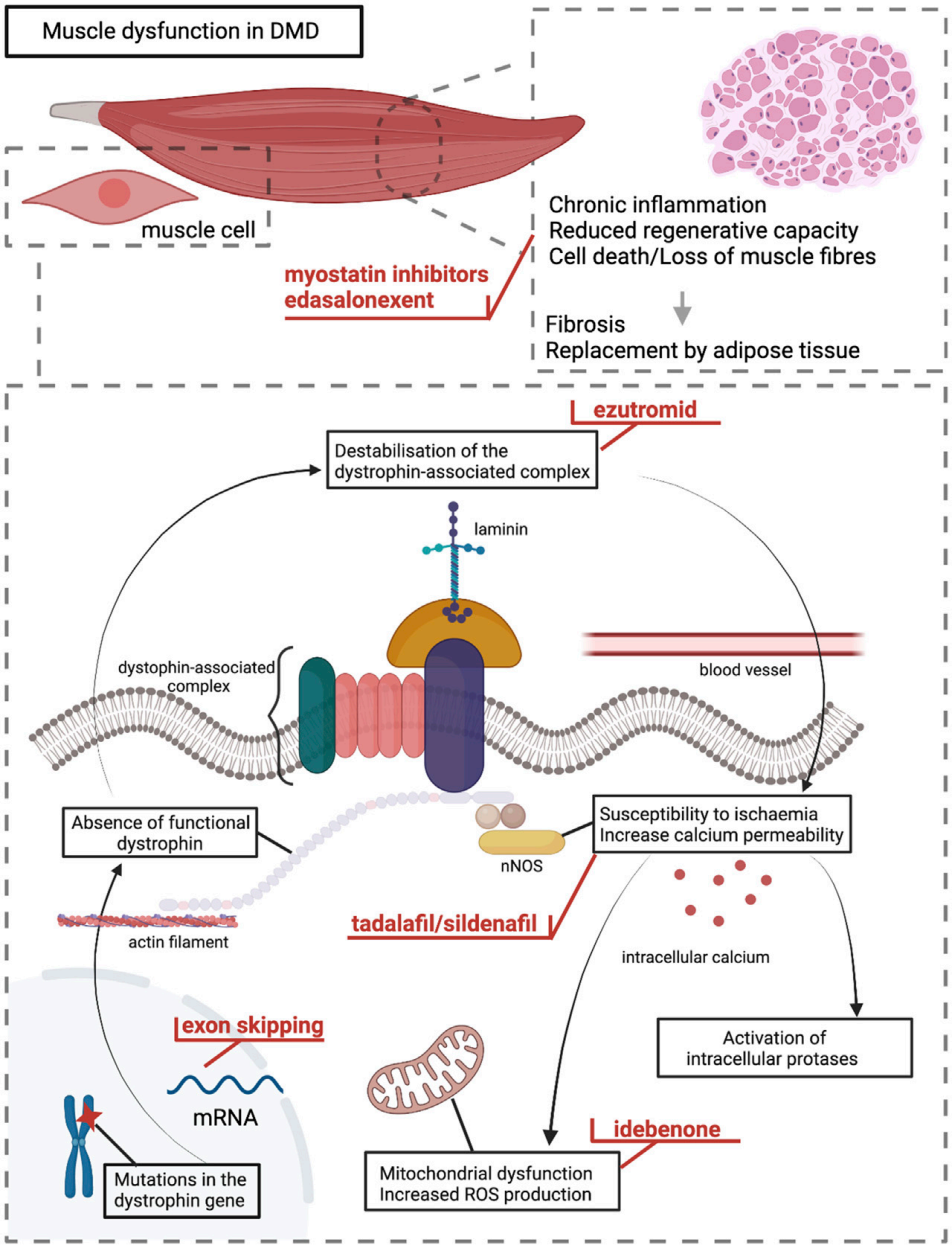
DMD pathophysiology and levels of action for the different compounds discussed in the paper. Mutations in the dystrophin gene lead to absence of functional dystrophin protein in muscle cells and destabilisation of the dystrophin-associated complex at the sarcolemma. This leads to increased susceptibility of the membrane to mechanical stress and ischaemia leading to increased membrane permeability, disturbed cellular signalling, and higher intracellular calcium levels causing activation of intracellular proteases. Mitochondrial dysfunction and increased production of reactive oxygen species contribute to DMD pathophysiology. These trigger a cycle of cell necrosis, chronic inflammation, and decreased muscle regenerative capacity. Ultimately, muscle fibres are lost and replaced by connective and adipose tissue resulting in muscle dysfunction. The different levels of action for the compounds discussed in the paper are indicated in red colour.

By searches of relevant databases, we identified 16 compounds that had been in clinical development in the DMD population (Table 1). Overall, compounds aiming to treat DMD can be classified into two broad categories: upstream treatments and downstream treatments. Upstream treatments aim to treat the cause of the disease, namely the deficiency of functional dystrophin. This category includes the therapeutic approaches of gene transfer therapy as well as exon skipping and stop codon readthrough. The two latter are highly mutation-specific. Downstream treatments target different levels of DMD pathophysiology. This therapeutic category applies to all DMD mutations and potentially to other conditions with shared pathophysiology such as Becker muscular dystrophy or limb-girdle muscular dystrophies. The Figure 1 is a schematic presentation of DMD pathophysiology on which the different levels of action of the discontinued clinical developments are demonstrated.

**TABLE 1:** Compounds discontinued during clinical development for DMD.

Compound	Mechanism	Route	Phase	N	Cohort age	Primary efficacy endpoint	Reason of discontinuation	Signal of efficacy	Identifier (clinicaltrials.gov)
drisapersen/PRO051/BMN051/GSK2402968 (or Kyndrisa)	skipping of exon 51	SC	III	186	≥5	6MWT	safety/efficacy	improvements in 6MWT in population with a baseline 6MWT of 300–400 m	NCT01254019
PRO044/BMN044	skipping of exon 44	IV/SC	I/II	18	5–16	safety, dystrophin expression	linked with drisapersen	-	NCT01037309
PRO045/BMN045	skipping of exon 45	SC	I/II	15	5–18	6MWT	linked with drisapersen	-	NCT01826474
PRO053/BMN053	skipping of exon 53	IV/SC	I/II	9	5–18	6MWT	linked with drisapersen	-	NCT01957059
suvodirsen/WVE-210201	skipping of exon 51	IV	II/III	6	5–12	dystrophin expression, NSAA	PK	-	NCT03907072
domagrozumab/PF-06252616	myostatin inhibition	IV	II	121	6–15	safety, 4SC	efficacy	increased muscle mass, improvement on NSAA	NCT02310763
ACE-031	myostatin inhibition	SC	II	24	≥4	safety	safety	increased lean body mass, improvement on 6MWT	NCT01099761
talditercept alfa/RG6206/ RO7239361	myostatin inhibition	SC	II/III	166	6–11	NSAA	efficacy	increased lean body mass	NCT03039686
tadalafil	vasodilation	oral	III	331	7–14	LVESV on CMR imaging	efficacy	decreased decline on PUL in older subjects	NCT01865084
sidenafil	vasodilation	oral	II	20*	18–50	FVC %predicted	safety	-	NCT01168908
idebenone	Q10 analogue/antioxidant action	oral	III	256	≥10	FVC	efficacy	improved respiratory function in steroid-naïve population	NCT02814019
ezutromid/SMT C1100	utrophin upregulation	oral	II	40	≥5	MRI-T2 and fat fraction for leg muscles	efficacy/PK	improved parameters on imaging, increased utrophin levels on biopsies	NCT02858362
edasalonexent/CAT-1004	NF-kB inhibitor/anti-inflammatory action	oral	III	131	4–7	safety, NSAA	efficacy	improvements in NSAA in younger population	NCT03703882
MNK-1411/cosyntropin	anti-inflammatory action	SC	II	44	4–8	10 MW/R	slow recruitment	-	NCT03400852
HT-100/halofuginone	anti-inflammatory/anti-fibrotic action	oral	I/II	17	6–20	safety	safety	-	NCT01847573
P-188NF	membrane stability	SC	II	10	12–25	FVC	drug supply	-	NCT03558958

*including patients with Becker muscular dystrophy.

CMR, cardiac magnetic resonance; SC, subcutaneous; IV, intravenous; 6MWT, 6-Minute Walk Test; NSAA, North Star Ambulatory Assessment; 4SC, 4 Stair Climb Time; LVESV, left ventricular end-systolic volume; FVC, forced vital capacity; MRI-T2, transverse relaxation time on MRI; 10MW/R, 10 Meter Walk/Run; PK, pharmacokinetics.

### Compounds Targeting Specific DMD Mutations

The goal of exon-skipping approaches, which are mutation-specific, is modulation of splicing of the *dystrophin* pre-mRNA to restore the reading frame in cases of frameshift mutations (deletions or duplications) which result in truncated dystrophin protein. Two ASOs that induce exon 51 skipping (applicable to approximately 14% of patients) have failed in late-phase trials. One of these is drisapersen, an ASO modified with 2′-*O*-methyl and phosphorothioate that showed promising preclinical results (van Deutekom et al., 2001; Lu et al., 2005; Alter et al., 2006; McClorey et al., 2006). Drisapersen was assessed in an open-label extension of a dose-finding phase I trial that included 12 DMD patients. Participants who were able to complete 330 m or more in the 6-Minute Walking Test (6MWT), a functional assessment of mobility, at baseline showed remarkable improvement after 177 weeks. However, two patients who had a baseline 6MWT of less than 330 m lost ambulation (Goemans et al., 2016). Drisapersen was tested in two phase II and a phase III, placebo-controlled trials of approximately 300 DMD patients. In the first phase II (DMD114117; NCT01153932) trial 53 participants (≥5 years) received continuously (weekly) or intermittently (combination of twice weekly and weekly administration, followed by no administration periods) placebo or drisapersen at 6 mg/kg for 48 weeks. A key inclusion criterion was the ability to raise from the floor in less than 7 s, which is a strong positive prognostic factor for maintaining ambulation over the next 2 years (Mazzone et al., 2016). There was a statistically significant improvement on the 6MWT (primary outcome) for continuously treated patients when compared with the placebo group at week 25 (study midpoint) but not for the intermittently treated group. Analysis of the population of the two subgroups suggested that drisapersen might benefit younger patients at an earlier stage of the disease more than older subjects. In the second phase II (DMD114876; NCT01462292) trial, the 51 participants (≥5 years) were randomised to placebo, 3 mg/kg/week drisapersen or 6 mg/kg/week drisapersen. The placebo and the low-dose group showed a decline in the 6MWT at 24 weeks (primary endpoint), whereas the high-dose group improved; however, this difference was not statistically significant (Voit et al., 2014; McDonald et al., 2018a). The randomised, placebo-controlled phase III (DMD114044; NCT01254019) trial involved 186 participants (≥5 years) with a later stage disease and who were on background treatment with steroids. Lack of inclusion criteria on the time-to-rise from floor test translated into the inclusion of patients who lost ambulation during the trial period. As the number of patients was much larger than in the phase II studies, broader inclusion criteria were needed as were a larger number of investigation sites. Ultimately, the difference between drisapersen-treated (with 6 mg/kg/week) and placebo-treated patients did not reach statistical significance on the 6MWT at 48 weeks or on key secondary endpoints. Only the *post-hoc*-defined subgroup of patients scoring between 300 and 400 m on the 6MWT at baseline significantly benefited from therapy. The occurrence of adverse events including injection site reactions, thrombocytopenia, and proteinuria in combination with the lack of efficacy led to the termination of the clinical development of this compound (Goemans et al., 2018).

The company developing drisapersen was also developing ASOs PRO044, PRO045, and PRO053 designed to induce exon 44, exon 45 and exon 53 skipping, respectively. Development of these compounds was discontinued at the same time drisapersen development was halted. The development of drisapersen demonstrated that it is crucial to recruit a large, representative sample of patients because of the heterogeneity in the disease trajectory. Because of the rarity of the disease, this is very challenging, leading to broad inclusion criteria that further increases heterogeneity. Moreover, the need to include a big number of sites considerably increases the variability in standards of care and data collection.

Suvodirsen was another ASO designed to induce exon 51 skipping. Suvodirsen is a stereopure ASO, which was expected to result in a better pharmacodynamic and pharmacokinetic profile, that would translate into improved safety and efficacy. Suvodirsen has shown very promising results *in vitro*; however, in a phase II/III (DYSTANCE 51; NCT03907072) trial, suvodirsen failed to increase dystrophin expression as assessed by western blot in muscle biopsies; its development was stopped due to inability to show target engagement (Sun et al., 2020).

### Compounds Applicable to all DMD Mutations

#### Myostatin Inhibitors

Myostatin is a member of the transforming growth factor-beta family, and its levels are negatively associated with muscle growth. Myostatin inhibition has long been considered a promising intervention to reverse pathology in muscle-wasting diseases, and in preclinical studies decreased myostatin levels lead to increased muscle mass and strength as well as decreased fibrosis (Wagner et al., 2002; McCroskery et al., 2005; Wagner et al., 2005). Three myostatin inhibitors have been tested in DMD patients in randomised, placebo-controlled trials including the monoclonal neutralising antibody domagrozumab, the soluble ActRIIB ligand ACE-031, and talditercept alfa, which is an adnectin anti-myostatin protein. In a randomised, placebo-controlled phase II (NCT02310763) trial of 121 patients (6–15 years), domagrozumab-treated patients at three different doses did not differ significantly from placebo-treated patients in the 4-Stair Climb (4SC) assessment at 49 weeks (primary endpoint). Development of domagrozumab was discontinued despite the fact that treated participants showed a trend of increasing muscle mass volume, assessed by MRI, and improvement on the North Star Ambulatory Assessment (NSAA) score (Wagner et al., 2020). ACE-031 injection in a randomised, placebo-controlled phase II (NCT01099761) trial of 24 patients (≥4 years) caused dose-dependent increases in lean body mass and improvement in 6MWT; however, spontaneous bleeding associated with angiogenetic action of ACE-031 was observed. Due to safety reasons, the development was terminated (Campbell et al., 2017). In a phase I trial, talditercept alfa had a good safety profile and induced an increase in the lean body mass of participants. A phase II (NCT03039686) trial in 166 ambulant participants (6–11 years) was discontinued based on the prediction that a significant change in NSAA score (primary endpoint) at 48 weeks would not be achieved (Wagner et al., 2019).

There has been a failure to translate positive results of myostatin inhibition in animal models to humans. Myostatin is downregulated in muscle-wasting conditions in humans and dystrophin-deficient *mdx* mice. However, the myostatin levels in *mdx* mice remain much higher (25% of wild-type levels) than in humans with DMD (8% of normal values). Thus, further inhibition of myostatin in humans may not have the therapeutic effect seen in mice. In addition, myostatin is naturally downregulated in many neuromuscular diseases. Of note, myostatin inhibitors showed signals of efficacy as assessed by the lean/muscle mass with non-invasive imaging, but failed to show effect on functional outcomes (domagrozumab, talditercept alfa) or raised safety concerns (ACE-031) (Mariot et al., 2017; Koch et al., 2020; Rybalka et al., 2020; Wagner, 2020).

#### Tadalafil and Sildenafil

Repurposing compounds with a known safety and tolerability profile for rare diseases can accelerate approval processes. A case of repurposing was that of the phosphodiesterase type 5 inhibitors, tadalafil and sildenafil, which are approved for use in erectile dysfunction and pulmonary arterial hypertension. Dystrophin deficiency leads to decreased levels of the muscle-specific neuronal nitric oxide (NO) synthase at the sarcolemma and subsequently decreased production of NO. The NO-cyclic guanosine monophosphate pathway is involved in protective functional sympatholysis, a mechanism by which muscle blood flow increases with activity, to provide the muscle with oxygen and nutrients. Disruption of this protective mechanism exacerbates DMD pathology by making muscles prone to activity-induced ischemia and by increasing production of reactive oxygen species (Timpani et al., 2017; Timpani et al., 2020). The hypothesis that phosphodiesterase inhibition would increase muscle blood flow was supported by preclinical data in *mdx* mice (Percival et al., 2012; Nio et al., 2017). However, neither compound showed efficacy during clinical assessment (Timpani et al., 2020). The development of sildenafil was terminated earlier than that of tadalafil due to cardiomyopathy worsening observed in some of the treated participants in a placebo-controlled phase II (REVERSE DBMD; NCT01168908) trial, which also included patients affected by Becker muscular dystrophy (Leung et al., 2014). Assessment of tadalafil progressed to a randomised, placebo-controlled phase III (NCT01865084) trial in 331 participants (7–14 years) with a background treatment of steroids in which treated participants received either 0.3 mg/kg/d or 0.6 mg/kg/d tadalafil for 48 weeks. Tadalafil failed to show efficacy, as assessed by the 6MWT, which declined faster in the treated groups (Victor et al., 2017). Of note, these trials included one of the largest numbers of participants.

#### Idebenone

Another disappointing story of drug repurposing is that of the antioxidant Q10 analogue idebenone, which showed promising preclinical results in the *mdx* mouse model (Buyse et al., 2009). In a proof-of-concept randomised, placebo-controlled phase II trial, which included 21 patients (8–16 years) and lasted 12 months, it was demonstrated that idebenone slowed the loss of respiratory function in steroid-naïve subjects (Buyse et al., 2013). A randomised, placebo-controlled phase III (DELOS; NCT01027884) trial that included 64 steroid-naïve patients (10–18 years) with change in peak expiratory flow (PEF) from baseline to week 52 of treatment as primary endpoint reported significant effect in favour of the patients treated with idebenone (900 mg/d) (Buyse et al., 2015), which was sustained when patients were followed for up to 6 years through the Expanded Access Program (Servais et al., 2019). However, the primary outcome of the study was not an approved endpoint at that time (Buyse et al., 2017; Mayer et al., 2017). Idebenone (900 mg/d) was further assessed in a randomised, placebo-controlled, phase III (SIDEROS; NCT02814019) trial in 256 participants (≥10 years), who were not steroid-naïve and who were in the respiratory decline phase of the disease. Early interim analysis suggested that the study was unlikely to meet its primary endpoint (FVC% predicted) at 18 months. The development of idebenone was terminated despite earlier signs of efficacy in improving respiratory function in steroid-naïve patients.

#### Ezutromid

Ezutromid is an aryl hydrocarbon receptor antagonist that upregulates the production of utrophin, a dystrophin paralogue, and reverses DMD pathology in *mdx* mice (Tinsley et al., 1998). Ezutromid was overall safe and well-tolerated in early clinical development (Tejura et al., 2017; Muntoni et al., 2019a). A randomised, placebo-controlled phase II (PhaseOut DMD; NCT02858362) trial of two different ezutromid formulations (F3: microfluidised aqueous suspension, F6: powder suspension) included 40 participants (≥5 years), who were on stable steroid treatment. The primary endpoints were the fat fraction and the transverse relaxation time T2 measured by magnetic resonance imaging (MRI-T2) to assess muscle structure and inflammation levels at 12-weeks intervals. As secondary endpoints, the levels of utrophin and heavy chain myosin on muscle biopsies, as well as other functional outcomes were used (Muntoni et al., 2017). An interim analysis at 24 weeks based on the results from imaging and the muscle biopsies was positive, but at 48 weeks improvement was not seen. The lack of clinical efficacy signs combined with the challenging pharmacokinetics that ezutromid showed in earlier phases of development led to the cessation of its development (Wilkinson et al., 2019).

#### Edasalonexent

In DMD patients, the NF-kB pathway is activated, which is associated with inflammation and muscle degradation. The potent anti-inflammatory action of NF-kB inhibitors justified clinical testing as an alternative to the long-term use of steroids, which is linked to serious adverse effects (McDonald et al.,2018b). One of these NF-kB inhibitors, edasalonexent was safe, well-tolerated, and had good target engagement, as assessed by its ability to decrease NF-kB downstream pathways after 1 week of treatment in a phase I trial (Finanger et al., 2019). In a three-part phase I/II (MoveDMD; NCT02439216) trial, 31 participants (4–7 years), not on treatment with steroids were enrolled and the primary endpoint was the MRI-T2 of 5 lower leg muscles at 12 weeks. The primary endpoint was not met in the pooled analysis of the two edasalonexent doses (67 mg/kg/d, 100 mg/kg/d). This was attributed to the short observation period and the small sample size combined with the phenotypic variability. However, for the highest dose (100 mg/kg) MRI-T2 was improved compared to the off-treatment period after 12-weeks of treatment, with a good safety profile (Finkel et al., 2021a). Therefore, edasalonexent progressed in the subsequent randomised, placebo-controlled phase III (PolarisDMD; NCT03703882) trial, which enrolled 131 boys (4–7 years), who had been on steroids for at least 6 months, in which the primary outcome was not met. More specifically, there was no significant difference between the treated and the placebo group in the NSAA score (primary outcome) after 12 months of treatment with 100 mg/kg/d edasalonexent. Additionally, none of the secondary outcomes was met. Younger patients showed a non-significant one-point difference in the NSAA score compared to the placebo group, and a significant difference on secondary endpoints, including the 4SC assessment and timed function tests. However, when considering the overall population, the one-year change was similar between the two groups for all endpoints. This led to the discontinuation of the clinical development (Finkel et al., 2021b). Other NF-kB inhibitors (e.g., vamorolone, flavocoxid) are still in clinical development.

## Discussion

Despite almost three decades of preclinical and clinical research, no treatment other than steroids has formally demonstrated clinical efficacy in DMD. The attrition rate between early-phase and late-phase development is high (Dovari et al., 2021). Several developments have been discontinued due to inefficacy despite early-phase positive results or signals of efficacy in secondary outcomes. The pressure by investors and by stakeholders to present and publish positive results has probably contributed to an inflation of confirmatory trials with compounds that are unlikely to gain approval. Currently, several compounds are in clinical development for DMD, with five of them having received approval subject to further proof of efficacy. There is a clear need for better clinical trial design and more reliable outcome measures. Reasons for failures and suggested solutions are summarised in Table 2.

**TABLE 2:** Reasons for termination and suggested solutions.

Reasons for termination	Suggested solutions
1. Discrepancy in results between preclinical and clinical research Leung et al. (2014); Victor et al. (2017); Rybalka et al. (2020); Wagner. (2020); Soblechero‐Martín et al. (2021)	Larger animals with no difficulties to breed Larcher et al. (2014)
	Animal models representative of the human disease Larcher et al. (2014)
	Less inter-animal variability Larcher et al. (2014)
2. Disease rarity: insufficient sample sizes and insufficient exposure time Goemans et al. (2018); Finkel et al. (2021b)	Sensitive and objective outcome measures Hogrel et al., (2016); Lilien et al. (2019); Ropars et al. (2020)
	Digital or lab-based biomarkers Haberkamp et al. (2019); Lilien et al. (2019)
	Sharing of placebo data Goemans et al. (2020a)
	Enrichment of placebo arms with data from natural history studies Mercuri et al. (2020)
3. Disease heterogeneity: non-linear disease progression and variability in response Ricotti et al. (2016); Goemans et al. (2018); Finkel et al. (2021b)	Disease modeling and use of Bayesian statistical design of clinical trials Rooney et al. (2014); Fouarge et al. (2021)
	Stratification of patients by disease trajectory Mercuri et al. (2016); Muntoni et al. (2019b); Goemans et al. (2020b)
	Normalisation of data based on growth and developmental stage Goemans et al. (2013); Poleur et al. (2021)
	Refinement of inclusion criteria based on predictors of disease trajectory Mercuri et al. (2016)
4. Insufficient characterisation of surrogate endpoints Servais et al. (2019)	Characterisation of relationships between surrogate endpoints and functional outcomes Le Guiner et al. (2017); de Feraudy et al. (2021)
	Characterisation of time needed between observed differences in surrogate endpoints and observed differences in functional outcomes Servais et al. (2019); Servais et al.
5. Variability between investigation sites Goemans et al. (2018)	Evaluator training and strict protocols for procedures Mazzone et al. (2009)
	Objective and investigator-independent outcome measures Hogrel et al. (2016); Lilien et al. (2019); Ropars et al. (2020)

More extensive preclinical studies could contribute to more effective clinical trials; however, current preclinical evaluation modes are not optimal. The commonly used *mdx* mouse model has significant differences from patients affected by DMD, mainly because an accelerated muscle regeneration rate in mice results in milder symptoms (Egorova et al., 2021). The pathophysiology of the disease may also differ. For example, myostatin levels are higher in *mdx* mice. This likely resulted in differences in the efficacy of myostatin inhibitors in *mdx* mice and DMD patients. Additionally, confounding factors encountered in clinical trials such as the use of steroids are not an issue in preclinical studies. The recently reported dystrophin-deficient rat could allow more rigorous preclinical studies in a more relevant model and a larger number of animals, which would help tackle the issue of inter-animal variability (Larcher et al., 2014; Nakamura et al., 2015).

The variability of DMD genotypes results in a wide range of phenotypes. For example, in patients with certain point mutations, alternate splicing can lead to some production of functional dystrophin and consequently less severe symptoms. Other genetic modifiers, such as LTBP4, may also contribute to phenotypic variability (Barp et al., 2015; Hightower and Alexander, 2018; Bello et al., 2020). Furthermore, differences in standards of care may result in significant differences in the disease trajectory. The age at which steroids are started, the type of steroids used (deflazacort vs. prednisone) as well as the steroid regimen (e.g., daily vs. alternate days vs. 10 days on -10 days off) also influence disease trajectory (Schreiber et al., 2016; de la Peña et al., 2017; Pradhan and Das, 2018). Additionally, the disease follows a non-linear trajectory. Loss of ambulation occurs between 6 and 18 years of age, which leads to significant variability in phenotype around the age of 7, the typical age of a patient included in a clinical trial. In children between 4 and 7 years, motor function outcomes used in clinical trials, such as the NSAA score, might show development-associated improvement before declining due to the natural course of the disease (Goemans et al., 2013; Mcdonald et al., 2013; Ciafaloni et al., 2016; Ricotti et al., 2016). For another example, respiratory outcomes may improve due to the normal growth of patients leading to increased lung volumes. Furthermore, it is highly important to normalise data for both age and height in this population, as some DMD patients have significantly stunted growth, mainly due to chronic steroid treatment (Hogrel et al., 2020). With regards to clinical trial design, the need for larger samples and longer duration is hampered by the rarity of the disease. Additionally, the regulatory pressure for the use of placebo arms contradicts the wishes of the community (Peay et al., 2018). The stratification of patients by disease trajectory (Muntoni et al., 2019b) to better define inclusion criteria, the use of natural history data to enrich placebo arms (Bello et al., 2016; Goemans et al., 2020a; Mercuri et al., 2020; Osorio et al., 2020), the sharing of placebo data *via* platforms (Bello et al., 2016; Goemans et al., 2020a), the use of innovative and sensitive outcome measures (Seferian et al., 2015; Lilien et al., 2019), or the use of Bayesian modelling could all result in more efficient and informative clinical trials (Fouarge et al., 2021).

There is a significant unmet need with regards to outcome measures used in clinical trials; different endpoints are meaningful depending on the age and disease stage of DMD patients. The use of surrogate endpoints, such as dystrophin expression for the compounds which induce dystrophin production, require further investigation, as the extent to which these measures predict efficacy is unknown (Aartsma-Rus and Arechavala-Gomeza, 2018). The optimisation of dystrophin quantification methods with the aim to improve accuracy and reproducibility will provide an undeniable pharmacodynamic endpoint for clinical trials. Towards this step, the harmonisation of protocols of the most used assays (e.g., immunochemistry, western blot) for the quantification and localisation of dystrophin will help minimise intra- and inter-lab variability. Several steps are currently being taken towards the optimisation of current assays and the use of new technologies which will help the reliable and accurate dystrophin quantification (Wells, 2019). However, there are different arguments for the clinical relevance of low levels of dystrophin; some patients who produce low dystrophin levels from birth show a milder phenotype, but it is still under investigation if low levels can have the same result after years of disease evolution (de Feraudy et al., 2021). Additionally, dystrophin levels can be variable in healthy individuals (Aartsma-Rus et al., 2019).

The FDA has granted accelerated approval based on truncated dystrophin expression as a surrogate endpoint, whereas the EMA has been consistently requiring functional data (Aartsma-Rus and Arechavala-Gomeza, 2018). This more conservative attitude translates into a clear controversy between the two agencies when it comes to drug approvals. Given the burden of the intravenous infusion, the risk of infections associated with the use of port-a-cath, the potential side effects as well as the cost of such drugs, the EMA position seems reasonable. Nevertheless, the difference in the position of the two agencies complicates clinical development, which must integrate both the possibility of accelerated approval and the need to prove efficacy by strong clinical data -sometimes even when this drug is commercially distributed in the US-. This is currently the case for exon skipping ASOs, like casimersen, golodirsen and viltolarsen, which are commercially distributed in the US, and still in large scale placebo-controlled trials. The qualification of dystrophin levels as a validated surrogate endpoint will be key in the next years; the translation of a limited re-expression of dystrophin into a sustainable clinical benefit could potentially not only support early approval but also help to compare compounds with a mechanism of action based on dystrophin re-expression. The minimum amount of dystrophin that is needed, the critical age or tissue quality at which re-expression must be initiated to be efficient and the time needed to observe clinically significant benefit is currently unknown. On the other side, functional outcome measures are very much dependent on both the observer’s opinion and the patient’s fatigue and motivation. The use of endpoints based on continuous monitoring with wearable technologies, such as the 95th percentile stride velocity, has the potential to dramatically decrease the number of patients needed as well as the study duration (Haberkamp et al., 2019; Poleur et al., 2021).

Now, breakthroughs in the fields of genetic therapies bring several opportunities in the preclinical and clinical pipeline of DMD, which need to be efficiently assessed in clinical trials. The rarity and variability of DMD render the design of clinical trials challenging. Undoubtedly, the DMD community needs to act to avoid repeating the mistakes of the past. The development of representative disease models for preclinical studies, the identification of sensitive biomarkers, the use of innovative technology, and the application of statistics will assist the roadmap to a more efficacious clinical trial design.

## Data Availability

The original contributions presented in the study are included in the article/Supplementary Material, further inquiries can be directed to the corresponding author.

## Footnotes

1
https://www.fda.gov/news-events/press-announcements/fda-approves-targeted-treatment-rare-duchenne-muscular-dystrophy-mutation.
