# Supplementary material for: Lessons Learned from Discontinued Clinical Developments in Duchenne Muscular Dystrophy
Source: Front Pharmacol. 2021 Nov 1;12:735912. doi: 10.3389/fphar.2021.735912 (PMC8591262; doi:10.3389/fphar.2021.735912)
Supplement: Supplementary file 1 [file Presentation1.pdf]

Supplementary Material

Search Summary

A) Ovid MEDLINE(R) ALL <1946 to April 22, 2021>

#	Searches
1	Muscular Dystrophy, Duchenne/
2	duchenne*.ti,ab,kf.
3	dmd.ti,kf.
4	dmd.ab. /freq=2
5	((pseudohypertrophic or pseudo hypertrophic) adj3 muscular dystroph*).ti,ab,kf.
6	((x link or x linked or xlink or xlinked or 3b) adj dilated cardiomyopath*).ti,ab,kf.
7	or/1-6
8	randomized controlled trial.pt.
9	controlled clinical trial.pt.
10	"clinical trial, phase i".pt.
11	"clinical trial, phase ii".pt.
12	clinical trial, phase iii.pt.
13	clinical trial, phase iv.pt.
14	double-blind method/
15	clinical trials as topic/
16	clinical trials, phase i as topic/
17	clinical trials, phase ii as topic/
18	clinical trials, phase iii as topic/
19	clinical trials, phase iv as topic/
20	randomized controlled trials as topic/
21	controlled clinical trials as topic/
22	("4 arm" or "four arm").ti,ab,kw.
23	((single or doubl* or tripl* or treb*) and (blind* or mask*)).ti,ab,kw.
24	(randomized or randomised).ab.
25	placebo.ab.
26	drug therapy.fs.
27	randomly.ab.
28	trial.ab.
29	groups.ab.
30	or/8-29
31	exp animals/ not humans.sh.
32	30 not 31

33	7 and 32
34	limit 33 to yr="2016 -Current"

B) Embase Classic+Embase <1947 to 2021 April 22>

#	Searches
1	*Duchenne muscular dystrophy/
2	duchenne*.ti,ab,kw.
3	dmd.ti,kw.
4	dmd.ab. /freq=2
5	((pseudohypertrophic or pseudo hypertrophic) adj3 muscular dystroph*).ti,ab,kw.
6	((x link or x linked or xlink or xlinked or 3b) adj dilated cardiomyopath*).ti,ab,kw.
7	or/1-6
8	Randomized controlled trial/
9	Controlled clinical trial/
10	random$.ti,ab.
11	randomization/
12	intermethod comparison/
13	placebo.ti,ab.
14	(compare or compared or comparison).ti.
15	((evaluated or evaluate or evaluating or assessed or assess) and (compare or compared or comparing or comparison)).ab.
16	(open adj label).ti,ab.
17	((double or single or doubly or singly) adj (blind or blinded or blindly)).ti,ab.
18	double blind procedure/
19	parallel group$1.ti,ab.
20	(crossover or cross over).ti,ab.
21	((assign$ or match or matched or allocation) adj5 (alternate or group$1 or intervention$1 or patient$1 or subject$1 or participant$1)).ti,ab.
22	(assigned or allocated).ti,ab.
23	(controlled adj7 (study or design or trial)).ti,ab.
24	(volunteer or volunteers).ti,ab.
25	human experiment/
26	trial.ti.
27	or/8-26
28	(random$ adj sampl$ adj7 (cross section$ or questionnaire$1 or survey$ or database$1)).ti,ab. not (comparative study/ or controlled study/ or randomi?ed controlled.ti,ab. or randomly assigned.ti,ab.)
29	Cross-sectional study/ not (randomized controlled trial/ or controlled clinical study/ or controlled study/ or randomi?ed controlled.ti,ab. or control group$1.ti,ab.)
30	(((case adj control$) and random$) not randomi?ed controlled).ti,ab.

31 (Systematic review not (trial or study)).ti.

32 (nonrandom$ not random$).ti,ab.

33 Random field$.ti,ab.

34 (random cluster adj3 sampl$).ti,ab.

35 (review.ab. and review.pt.) not trial.ti.

36 we searched.ab. and (review.ti. or review.pt.)

37 update review.ab.

38 (databases adj4 searched).ab.

39 (rat or rats or mouse or mice or swine or porcine or murine or sheep or lambs or pigs or piglets or rabbit or rabbits or cat or cats or dog or dogs or cattle or bovine or monkey or monkeys or trout or marmoset$1).ti. and animal experiment/

40 Animal experiment/ not (human experiment/ or human/)

41 or/28-40

42 27 not 41

43 7 and 42

44 limit 43 to yr="2016 -Current"

C) Scopus

((TITLE-ABS-KEY (*duchenne**)) OR (TITLE (*dmd*)) OR (TITLE-ABS ((*pseudohypertrophic* OR *"pseudo hypertrophic"*) W/2 (*"muscular dystroph*"*))) OR (TITLE-ABS ((*"x link"* OR *"x linked"* OR *xlink* OR *xlinked* OR *3b*) W/1 (*"dilated cardiomyopath*"*)))) AND (TITLE-ABS-KEY (*{Clinical-trial}* OR *{controlled-trial}* OR *randomi** OR *randomly* OR (*random* W/4 (*allocat** OR *distribut** OR *assign**)) OR *{placebo}* OR *{trial}* OR *{groups}* OR *{subgroups}*) OR TITLE (*rct*)) AND (LIMIT-TO (PUBYEAR , *2021*) OR LIMIT-TO (PUBYEAR , *2020*) OR LIMIT-TO (PUBYEAR , *2019*) OR LIMIT-TO (PUBYEAR , *2018*) OR LIMIT-TO (PUBYEAR , *2017*) OR LIMIT-TO (PUBYEAR , *2016*))

D) Cochrane

ID Search Hits
#1 MeSH descriptor: [Muscular Dystrophy, Duchenne] this term only 228
#2 (duchenne*):ti,ab,kw 765
#3 ((pseudohypertrophic or "pseudo hypertrophic") AND (muscular dystroph*)):ti,ab,kw 1
#4 (("x link" or "x linked" or xlink or xlinked or 3b) AND (dilated cardiomyopath*)):ti,ab,kw 4
#5 #1 OR #2 OR #3 OR #4 with Publication Year from 2016 to 2021, with Cochrane Library publication date Between Jan 2016 and Apr 2021, in Trials 281
